# Popular Skin-of-Color Dermatology Social Media Hashtags on TikTok From 2021 to 2022: Content Analysis

**DOI:** 10.2196/50408

**Published:** 2024-10-18

**Authors:** Jeemin Kang, Mindy D Szeto, Lois Suh, Jadesola T Olayinka, Robert P Dellavalle

**Affiliations:** 1 Arizona College of Osteopathic Medicine, Midwestern University, Glendale, AZ, United States; 2 Department of Dermatology, University of Minnesota Medical School, 1-411 Phillips-Wangensteen Building, 516 Delaware St SE, MMC 98, Minneapolis, MN, 55455, United States, 1 612-625-8625; 3 Kirk Kerkorian School of Medicine, University of Nevada, Las Vegas, NV, United States; 4 Department of Dermatology, New York University Grossman School of Medicine, New York, NY, United States

**Keywords:** dermatology, dermatologist, social media, TikTok, skin of color, hashtag, content analysis, education, influencers, diversity, inclusion, disparities

## Abstract

TikTok is a social media platform that can educate users about dermatology, but this longitudinal analysis of skin of color–related TikTok hashtags from 2021 to 2022 suggests that nondermatologist influencers continue to dominate content creation, highlighting the need for more participation from board-certified dermatologists to actively counter misinformation and address potential disparities in skin-of-color health care.

## Introduction

Health care providers can use social media platforms such as TikTok to communicate with and educate users globally. Rapid dynamic sharing of content can be personalized and adapted to user preferences on TikTok through artificial intelligence–enabled algorithms and the “For You” feed [1], and users can publicly interact with and amplify trending videos by posting side-by-side “duets.” New features have continued to grow user engagement and personalization such that TikTok became the most popular platform by daily time spent in 2022 [2]. However, previous studies have established that nondermatologist influencers are prominent creators of dermatology-focused TikTok content, potentially disseminating dangerous misinformation [3]. Nevertheless, social media resources are particularly important to patients with skin of color (SoC), as their health care providers may be unfamiliar with ethnic skin and hair [4]. Given the fast-evolving nature of social media, we conducted a longitudinal study to build upon previous work [5], examine SoC-related hashtags on TikTok from 2021 to 2022, characterize the content of popular posts, and discuss their emerging impact on SoC dermatology.

## Methods

### Overview

We compiled 61 SoC-related hashtags from multiple reputable sources, including SoC journal literature; SoC-focused, peer-reviewed social media research; and the Skin of Color Society [4,6,7]. Each hashtag was searched on TikTok in August 2022 and compared to our results from 2021 [5]. To mitigate possible algorithmic bias, a new TikTok account was created to conduct all hashtag searches within 24 hours. Each SoC-related hashtag’s popularity by total related post views was examined using TokAudit.io. The top-viewed and top-liked posts for each hashtag were identified, along with their sources, types of content, and levels of engagement (views, likes, and comments). Content sources included self-identified US board-certified dermatologists, estheticians, non-US physicians, other health care providers, patients, and influencers. Inclusion criteria required self-identification in the user’s TikTok profile or within the post, and posts lacking content relevant to the searched hashtag were excluded. Two independent raters with medical education and dermatology experience categorized each post’s content: “educational” if clearly disseminating medical information, “promotional” if advertising a service or product, and “personal” for all other content, with discrepancies resolved through consensus meetings.

### Ethical Considerations

All research was conducted in compliance with regulations for the protection of human subjects under 45 CFR 46.104(d)(4), utilizing existing publicly available data without requiring additional contact or permissions from content creators [8].

## Results

Before consensus meetings, content categorizations showed high interrater agreement (Cohen κ=0.785 for top-viewed posts and 0.694 for top-liked posts). Considering our 2021 findings, the top SoC-related hashtags by total related post views in 2022 continued to be #IngrownHair (5.7B views), #HairLoss (3.1B views), #Dandruff (1.9B views), #Vitiligo (1.8B views), and #Hyperpigmentation (1.1B views; Table 1). #SkinofColorDermatology, #BrownSkinMatters, and #PseudofolliculitisBarbae remained among the least popular hashtags with existing posts for both years. Notably, 29% (5/17) of hashtags with no related posts in 2021 garnered new posts in 2022, including #XerosisCutis (219.1K views) and #AcneKeloidalis (5.3K views). Paralleling 2021, only 24% (12/49) of top-viewed and 20% (10/49) of top-liked posts (Table 2) were from board-certified dermatologists, and all contained educational content. Non-US dermatologists and physicians and other providers generated 24% (12/49) of top-viewed and 20% (10/49) of top-liked posts. Estheticians created 22% (11/49) of top-viewed and 20% (10/49) of top-liked posts, mostly promotional content. The remainder comprised promotional and personal posts from influencers and patients. Among available top-viewed content in 2022, #IngrownHair retrieved the most-viewed post (77.8M views, board-certified dermatologist, educational), followed by #HidradenitisSuppurativa (77.6M views, esthetician, promotional) and #Dandruff (46.5M views, influencer, personal). The highest user engagement was driven by personal patient videos of #Vitiligo (10.7M likes and 118K comments) and #HairLoss (6.4M likes and 71.9K comments).

**Table 1.** Characteristics of top-viewed TikTok skin-of-color content in August 2022, sorted by content category and number of views.

Content category and skin-of-color TikTok hashtag	Top-viewed post					Total views of hashtagged posts, n
	Source	Views , n	Likes , n	Comments , n	Non-English language	
**Educational posts (n=16)**						
#IngrownHair	Board-certified dermatologist	77,800,000	5,300,000	14,100		5,700,000,000
#Psoriasis	Board-certified dermatologist	20,000,000	1,100,000	16,000		689,400,000
#SebaceousCyst	Board-certified dermatologist	11,800,000	490,000	3184		70,700,000
#AcanthosisNigricans	Board-certified dermatologist	10,300,000	988,900	9018		56,000,000
#SeborrheicDermatitis	Board-certified dermatologist	9,100,000	373,500	4004		46,800,000
#Melanoma	Board-certified dermatologist	4,500,000	292,500	2007		138,000,000
#TineaVersicolor	Board-certified dermatologist	4,500,000	111,400	1918		11,300,000
#SeborrheicKeratosis	Board-certified dermatologist	3,200,000	14,500	257		7,100,000
#MelasmaTreatment	Board-certified dermatologist	2,400,000	80,800	694		72,300,000
#KeloidScar	Non-US physician	1,300,000	46,500	836	✓	14,300,000
#AtopicDermatitis	Board-certified dermatologist	1,100,000	110,400	1692		3,200,000
#DermatosisPapulosaNigra	Board-certified dermatologist	305,500	8532	180		503,100
#XerosisCutis	Non-US dermatologist	193,400	2655	47	✓	219,100
#DissectingCellulitisoftheScalp	Board-certified dermatologist	58,300	232	5		59,300
#PseudofolliculitisBarbae	Non-US physician	8171	131	6	✓	30,100
#AcneKeloidalis	Non-US physician	5340	72	0	✓	5355
**Promotional posts (n=17)**						
#HidradenitisSuppurativa	Esthetician	77,600,000	1,300,000	9391		366,000,000
#Melasma	Non-US physician	28,400,000	1,100,000	20,500	✓	621,300,000
#Hyperpigmentation	Esthetician	17,600,000	1,300,000	3519		1,100,000,000
#IngrownHairs	Esthetician	17,400,000	442,500	7534		106,300,000
#Hirsutism	Esthetician	17,200,000	954,200	3544		307,400,000
#Vitiligo	Influencer	15,400,000	2,900,000	3034		1,800,000,000
#RazorBumps	Influencer	14,500,000	3,000,000	8164		135,400,000
#Eczema	Patient	12,400,000	1,700,000	9882		802,000,000
#TractionAlopecia	Patient	7,500,000	729,300	0		34,800,000
#HairBreakage	Patient	5,500,000	879,700	2604		71,300,000
#MelanomaCancer	Esthetician	733,500	72,600	450		9,700,000
#CCCA	Esthetician	445,300	59,000	755		2,100,000
#SkinofColorDoc	Non-US dermatologist	322,500	6724	123		1,000,000
#PostInflammatoryHyperpigmentation	Other provider (nurse practitioner)	146,100	8311	126		463,600
#TineaCapitis	Non-US dermatologist	37,000	1188	10		1,200,000
#SkinofColorCare	Esthetician	448	3	0		555
#Dyschromia	Esthetician	14	0	0		130
**Personal posts (n=16)**						
#Dandruff	Influencer	46,500,000	3,200,000	18,800		1,900,000,000
#HairLoss	Patient	28,900,000	6,400,000	71,900		3,100,000,000
#Keloid	Patient	12,600,000	751,200	20,800		199,400,000
#Folliculitis	Esthetician	9,400,000	1,600,000	1350		36,200,000
#Keloids	Patient	8,000,000	153,700	2125		66,600,000
#SkinofColor	Non-US physician	3,100,000	593,700	604		14,800,000
#DiscoidLupus	Patient	3,100,000	467,700	5416		8,200,000
#Pseudofolliculitis	Non-US physician	380,900	22,200	367	✓	511,300
#DPNRemoval	Esthetician	215,000	15,300	355		627,300
#Sarcoidosis	Influencer	164,300	35,600	177	✓	8,100,000
#BrownSkinMatters	Patient	4103	44	0		5310
#DissectingCellulitis	Patient	1635	48	9	✓	1635
#CentralCentrifugalCicatricialAlopecia	Esthetician	1560	30	1		11,600
#DiscoidLupusErythematosus	Other provider (veterinarian)	1126	327	0	✓	39,000
#NonmelanomaSkinCancer	Influencer	840	8	0	✓	841
#SkinofColorDermatology	Non-US dermatologist	722	5	0	✓	722
**No posts (n=12)**						
#AsteatosisCutis, #Dyspigmentation, #EthnicDerm, #EthnicDermatology, #FolliculitisPapillaris, #SkinofColorDerm, #SkinofColorDermatologist, #SkinOfColorSociety, #SOCderm, #SOCdermatologist, #SOCdermatology, and #TrichorrhexisNodosa	—^a^	—	—	—	—	—

a Not applicable.

**Table 2.** Characteristics of top-liked TikTok skin-of-color content in August 2022, sorted by content category and number of likes.

Content category and skin-of-color TikTok hashtag	Top-liked post					
	Source	Views, n	Likes, n	Comments, n	Non-English Language	Same as the top-viewed post
**Educational posts (n=13)**						
#IngrownHair	Board-certified dermatologist	77,800,000	5,300,000	14,100		✓
#AcanthosisNigricans	Board-certified dermatologist	10,300,000	988,900	9018		✓
#SebaceousCyst	Board-certified dermatologist	11,800,000	490,000	3184		✓
#SeborrheicDermatitis	Board-certified dermatologist	9,100,000	373,500	4004		✓
#TineaVersicolor	Board-certified dermatologist	4,500,000	111,400	1918		✓
#AtopicDermatitis	Board-certified dermatologist	1,100,000	110,400	1692		✓
#SkinofColorDoc	Board-certified dermatologist	266,300	34,600	295		
#SeborrheicKeratosis	Board-certified dermatologist	3,200,000	14,500	257		✓
#DermatosisPapulosaNigra	Board-certified dermatologist	305,500	8532	180		✓
#XerosisCutis	Non-US dermatologist	193,400	2655	47		✓
#DissectingCellulitisoftheScalp	Board-certified dermatologist	58,300	232	5		✓
#PseudofolliculitisBarbae	Non-US physician	8171	131	6		✓
#AcneKeloidalis	Non-US physician	5340	72	0		✓
**Promotional posts (n=19)**						
#RazorBumps	Influencer	14,500,000	3,000,000	8164		✓
#Eczema	Patient	12,400,000	1,700,000	9882		✓
#HidradenitisSuppurativa	Esthetician	77,600,000	1,300,000	9391		✓
#Hyperpigmentation	Patient	7,100,000	1,300,000	5677		
#Melasma	Non-US physician	28,400,000	1,100,000	20,500		✓
#HairBreakage	Patient	5,500,000	879,700	2604		✓
#TractionAlopecia	Esthetician	5,100,000	841,800	3951		
#IngrownHairs	Esthetician	7,200,000	523,300	1370		
#Dandruff	Influencer	2,000,000	352,700	2,782		
#Melanoma	Esthetician	2,700,000	349,400	758		
#MelasmaTreatment	Influencer	1,700,000	103,500	487	✓	
#MelanomaCancer	Esthetician	733,500	72,600	450		✓
#CCCA	Esthetician	445,300	59,000	755		✓
#KeloidScar	Patient	899,800	51,300	234		
#PostInflammatoryHyperpigmentation	Other provider (nurse practitioner)	146,100	8311	126		✓
#TineaCapitis	Non-US dermatologist	37,000	1188	10		✓
#CentralCentrifugalCicatricialAlopecia	Esthetician	1541	72	0		
#SkinofColorCare	Influencer	106	14	0		
#Dyschromia	Esthetician	14	0	0		✓
**Personal posts (n=17)**						
#Vitiligo	Patient	6,900,000	10,700,000	118,000		
#HairLoss	Patient	28,900,000	6,400,000	71,900		✓
#Hirsutism	Patient	15,000,000	2,000,000	21,200		
#Psoriasis	Influencer	8,000,000	1,700,000	10,100		
#Folliculitis	Esthetician	9,400,000	1,600,000	1350		✓
#Keloid	Patient	12,600,000	751,200	20,800		✓
#SkinofColor	Non-US physician	3,100,000	593,700	604		✓
#DiscoidLupus	Patient	3,100,000	467,700	5416		✓
#Keloids	Patient	7,700,000	396,100	2470	✓	
#Sarcoidosis	Influencer	164,300	35,600	177		✓
#Pseudofolliculitis	Non-US physician	380,900	22,200	367		✓
#DPNRemoval	Esthetician	215,000	15,300	355		✓
#DiscoidLupusErythematosus	Other provider (veterinarian)	1126	327	0		✓
#BrownSkinMatters	Influencer	341	55	9		
#DissectingCellulitis	Patient	1635	48	9		✓
#NonmelanomaSkinCancer	Influencer	840	8	0		✓
#SkinofColorDermatology	Non-US dermatologist	722	5	0		✓
**No posts (n=12)**						
#AsteatosisCutis, #Dyspigmentation, #EthnicDerm, #EthnicDermatology, #FolliculitisPapillaris, #SkinofColorDerm, #SkinofColorDermatologist, #SkinOfColorSociety, #SOCderm, #SOCdermatologist, #SOCdermatology, and #TrichorrhexisNodosa	—^a^	—	—	—	—	—

a Not applicable.

## Discussion

Hashtags for hair and pigmentary disorders common in SoC such as #HidradenitisSuppurativa and #Hyperpigmentation remained popular in 2022, reflecting emerging societal attention toward sociocultural diversity and health disparities [9], along with growing SoC representation among social media content creators [10]. However, our study was limited by content that was not strictly SoC related but listed multiple hashtags, non–English-language posts, and a need for in-depth qualitative content analysis in future work. The lack of provider credential verification also posed barriers. Nevertheless, self-identified, board-certified dermatologists posted educational content as expected, garnering views and engagement comparable to promotional and personal posts from other sources, but they continued to comprise a small fraction of popular TikTok content generators. We reiterate our call for additional dermatologist engagement, sharing compelling patient stories while dispelling health misinformation. TikTok’s unique features could be leveraged to further boost influence.

## Abbreviations

SoC: skin of color
